# Pulmonary arterial hypertension in an 80-year-old man with long-term use of cyclophosphamide

**DOI:** 10.1016/j.rmcr.2023.101867

**Published:** 2023-05-11

**Authors:** Takatoshi Suzuki, Ichizo Tsujino, Wataru Harabayashi, Hideki Shima, Junichi Nakamura, Takahiro Sato, Masaru Suzuki, Yukari Takeda, Satohi Konno

**Affiliations:** a Department of Respiratory Medicine, Faculty of Medicine, Hokkaido University, N15, W7, Kita-ku, Sapporo, 060-8638, Japan; b Department of Hematology, Tonan Hospital, N4, W7, Kita-ku, Sapporo, 060-0004, Japan

**Keywords:** Primary macroglobulinemia, Cyclophosphamide, Pulmonary arterial hypertension

## Abstract

An 80-year-old man diagnosed with primary macroglobulinemia 7 years earlier had been treated with cyclophosphamide, following which he developed dyspnea on exertion. Cyclophosphamide was discontinued. The patient's dyspnea, however, failed to improve. Right heart catheterization (RHC) revealed precapillary pulmonary hypertension (PH). He was transferred to our institution for further examination. Prior use of cyclophosphamide was the patient's only risk factor for PH, and cyclophosphamide use was considered as a possible cause of PH in this case. He was treated with tadalafil and dyspnea gradually improved. A follow-up RHC exhibited improvement in mean pulmonary arterial pressure and pulmonary vascular resistance.

## 1 Introduction

Pulmonary arterial hypertension (PAH) develops after exposure to certain drugs and toxins [1]. In the current guidelines for pulmonary hypertension (PH), more than 20 drugs/toxins are listed as substances that may be associated with PAH [2]. These drugs are used under various clinical conditions. It is thus important for clinicians to recognize the drugs that can cause PAH and how drug-induced PAH develops and evolves in order to diagnose the disease early and provide optimal management.

Cyclophosphamide is an alkylating agent that is widely used in the treatment of various diseases, including hematologic malignancies and collagen diseases. It is known to induce cardiovascular adverse events in some cases and is included in pharmacologic agents with possible associations with PAH [2]. To date, several publications have reported cases of patients who developed PH associated with the use of cyclophosphamide [[3], [4], [5], [6], [7], [8]]. However, all these cases involved factors other than cyclophosphamide use that might have contributed to the development of PH; some were diagnosed with PH without right heart catheterization (RHC), or with limited clinical and hemodynamic data [[3], [4], [5], [6], [7], [8]].

Herein, we present the case of an 80-year-old man who developed PAH after 7 years of cyclophosphamide treatment. The present report is unique in that the diagnosis of PAH was confirmed by a fully documented complete diagnostic work-up, including RHC. In this case, PAH clearly developed after the use of cyclophosphamide without other established PAH risks. The documentation of serial RHC data after cyclophosphamide discontinuation and the use of tadalafil were also chronicled extensively.

## 2 Case presentation

An 80-year-old man, a non-smoker, was diagnosed with primary macroglobulinemia in 2015 and treated with plasmapheresis and chemotherapy. He had been treated with two courses of dexamethasone, bendamustine, and rituximab following a diagnosis of macroglobulinemia, after which four courses of dexamethasone and bortezomib were prescribed. Bortezomib, dexamethasone, and cyclophosphamide were administered for 4 years before the treatment was switched to cyclophosphamide alone in 2019. His condition remained stable all throughout the course of treatment. Cyclophosphamide was administered at an approximate dose of 500 mg/week. A transthoracic echocardiography (TTE) performed in 2019 revealed normal findings. In 2022, the patient developed dyspnea on exertion, which worsened. Subsequently, cyclophosphamide was discontinued 2 months later. In spite of this, his symptoms failed to improve. A TTE suggested elevated pulmonary arterial pressure, which led to hospitalization. An RHC conducted 2 months after the cessation of cyclophosphamide revealed a mean pulmonary artery pressure (mPAP) of 48 mmHg, pulmonary artery wedge pressure (PAWP) of 13 mmHg, cardiac output (CO) of 6.15 L/min, cardiac index (CI) of 3.19 L/min/m^2^, and pulmonary vascular resistance (PVR) of 4.9 Wood units (WU), leading to a diagnosis of precapillary PH. The patient was then transferred to our hospital for further investigation.

On admission, the patient's World Health Organization functional class was III. The patient had no pertinent medical history other than primary macroglobulinemia and no family history of PH. Physical examination revealed a heart rate of 80/min, blood pressure of 126/60 mmHg. Arterial blood gas analysis in room air showed a pH of 7.439, PO_2_ of 71.1 mmHg, PCO_2_ of 33.2 mmHg, HCO_3_^−^ of 22.1 mmol/L, and A-aDO_2_ of 37.1 mmHg. Blood tests revealed Hb of 11.1 g/dL and mildly elevated brain natriuretic peptide (BNP) level of 72.3 pg/mL. Gene mutations related to PH, including biallelic mutations in *EIF2AK4*, were not detected.

**Fig. 1:**
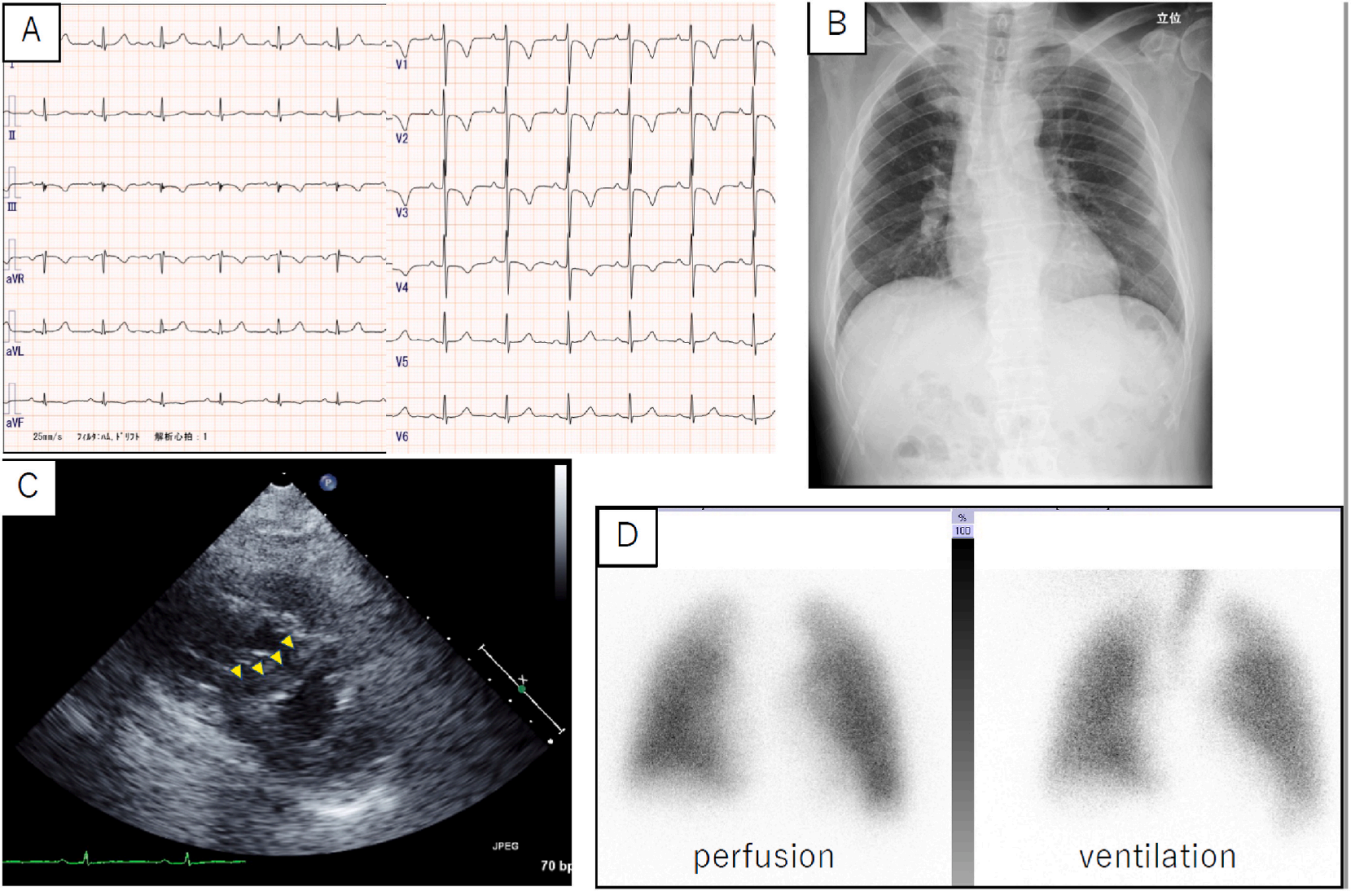
**A.** Electrocardiography shows a normal sinus rhythm with negative T waves in V1–4. **B.** The chest radiograph documents mild cardiomegaly and a dilated right pulmonary artery. **C**. Transthoracic echocardiogram reveals a dilated right ventricle along with compression of the left ventricle. **D**. Lung ventilation-perfusion scintigraphy shows no mismatch.

Electrocardiography revealed negative T waves in V1–4 (Fig. 1A). Chest radiography demonstrated mild cardiomegaly and dilated right pulmonary artery (Fig. 1B). TTE showed an elevated transtricuspid pressure gradient of 54 mmHg and a compressed left ventricle, forming a D-shape (Fig. 1C). Respiratory function tests showed severe diffusion impairment with a 10.9% predicted diffusing capacity of lungs for carbon monoxide (DLCO) despite the absence of restrictive or obstructive ventilatory impairment. Enhanced computed tomography (CT) did not detect pulmonary artery thrombi or any abnormalities in the lung field. Lung ventilation-perfusion scintigraphy revealed no mismatches (Fig. 1D).

**Fig. 2:**
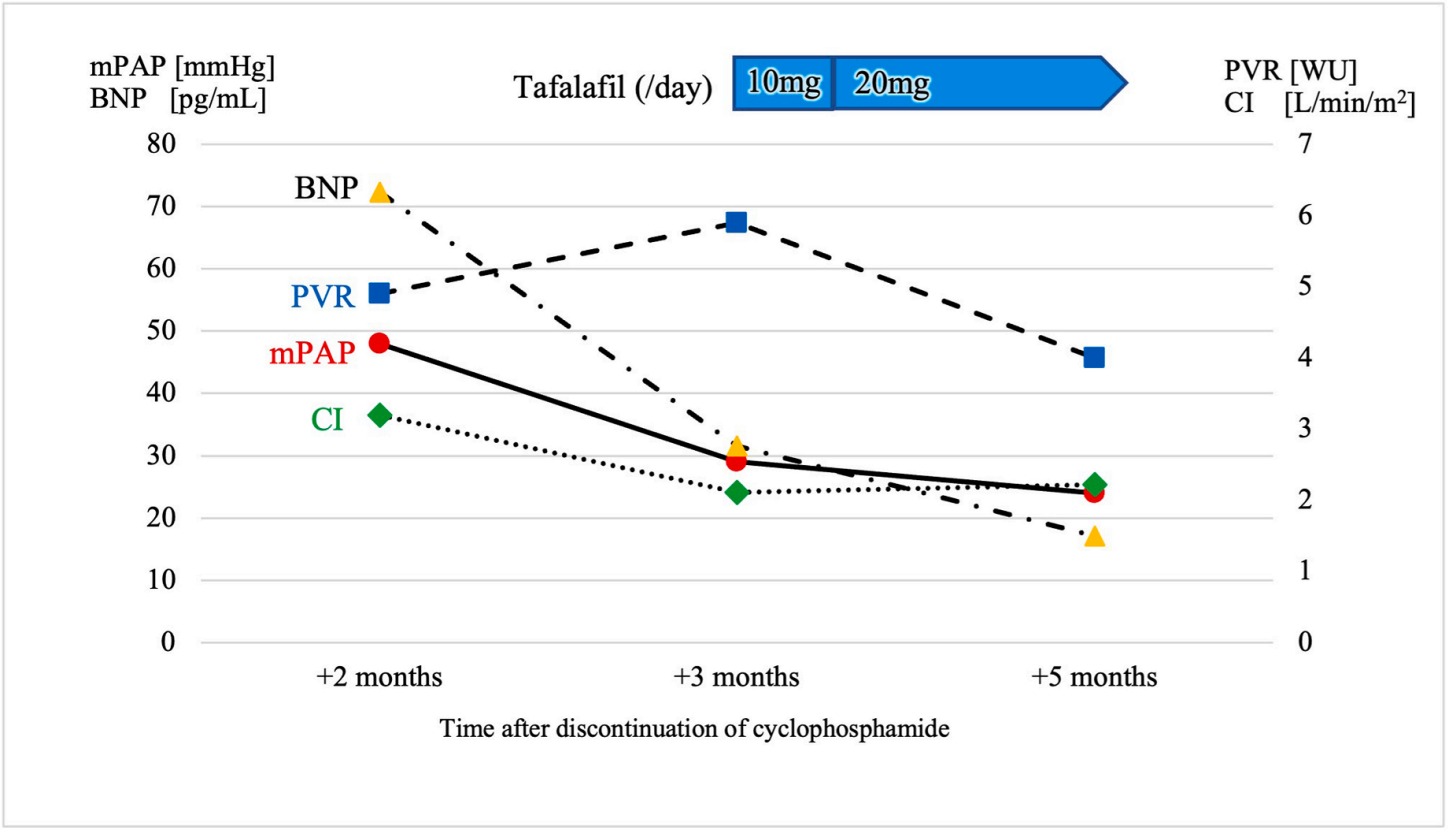
Trends in hemodynamics and plasma BNP concentration after the discontinuation of cyclophosphamide. BNP, brain natriuretic peptide; PVR, pulmonary vascular resistance; mPAP, mean pulmonary artery pressure; CI, cardiac index.

RHC performed at our hospital, 3 weeks after the previous examination, showed an mPAP of 29 mmHg, PAWP of 6 mmHg, CO of 3.9 L/min, CI of 2.11 L/min/m^2^ and PVR of 5.9 WU. The patient's body weight decreased by 2.6 kg compared to his body weight during the previous RHC. The decrease in mPAP, body weight, and PAWP suggested that fluid retention improved with salt reduction after hospitalization. However, PVR did not decrease. RHC data at this time indicated precapillary PH and, without any finding indicative of group 3, 4, or 5 PH; PAH was diagnosed accordingly. In addition, although the possibility of idiopathic PAH could not be excluded, cyclophosphamide-associated PAH was considered as a likely subtype of PAH, with cyclophosphamide use being the patient's only risk factor for PAH. Tadalafil was then started at 10 mg/day, and the dose was increased to 20 mg/day for 6 days. Subsequently, dyspnea gradually improved. RHC performed 2 months after treatment initiation showed hemodynamic improvement with an mPAP of 24 mmHg, PAWP of 8 mmHg, CO of 4.05 L/min, CI of 2.22 L/min/m^2^, and PVR of 4.0 WU (Fig. 2).

## 3 Discussion

### 3.1 Clinical discussion

To date, there have been approximately only 20 case reports, which have shown possible associations between cyclophosphamide use and PAH [[3], [4], [5], [6], [7], [8]]. However, only limited data were documented in these reports, and the association between the use of cyclophosphamide and the development of PAH was not necessarily evident. In the present report, compared to prior publications, more detailed clinical data are presented, including echocardiography before the development of PAH and DLCO, high-resolution computed tomography, and serial RHC after the cessation of cyclophosphamide.

In our case, the diagnosis of cyclophosphamide-associated PAH was most likely based on the patient's clinical course; he developed PAH after continuous cyclophosphamide use for 7 years, which was the patient's sole risk factor for PAH according to the current guidelines [2]. Notably, the patient underwent echocardiography 3 years ago and had unremarkable cardiopulmonary findings at the time. Upon receiving cyclophosphamide monotherapy after the discontinuation of other concurrent drugs, the patient subsequently developed PAH. He did not have any other risk factors for PAH, including connective tissue disease, portal hypertension, or congenital heart diseases. He also had no significant pulmonary or left heart disease or findings suggestive of chronic thromboembolic pulmonary hypertension on imaging studies. Hematological disorders are included as the causes of group 5 PH; however, currently, primary macroglobulinemia is not considered a risk factor for PH. It should also be noted that he had received three other chemotherapeutic drugs, bendamustine, rituximab, and bortezomib, up until 3 years before the development of PAH. However, the use of these three drugs were not considered risk factors for PAH. In addition, the >3-year interval between the discontinuation of these drugs and the development of PAH made the association between the use of these drugs and PAH less likely. However, there is no direct evidence that cyclophosphamide use caused PAH in our case. Thus, other possibilities, including idiopathic PAH that may coincidentally develop in an individual receiving cyclophosphamide, cannot be strictly excluded.

Among the subtypes of PAH, pulmonary veno-occlusive disease (PVOD) is the most frequently reported to be caused by cyclophosphamide use [4]. Pathological changes consistent with PVOD have also been reported in animal studies [4]. Thus, in our case, we also considered the possibility of PVOD. In our patient, respiratory function tests showed a remarkable decrease in DLCO of 10.9%, which is one of the hallmarks of PVOD [2]. However, there were no CT findings suggestive of PVOD, such as thickening of the interlobular septa, centrilobular opacities, mediastinal lymphadenopathy, or worsening of pulmonary congestion after the use of a pulmonary vasodilator. Therefore, PVOD was not clinically diagnosed in our patient. However, it is often difficult to clearly distinguish PAH from PVOD, and it is possible that a PVOD component was present in our case.

### 3.2 Brief review of literature

A systematic review reported by Benoit et al., in 2015 reviewed cases of chemotherapy-induced PVOD from the French PH network and reported that cyclophosphamide was used in 16 of the 37 cases they identified [4]. Aside from these 16 cases, there have been four other cases, in which PH developed following the use of cyclophosphamide [3,5,6]. Notably, in all these cases, multiple chemotherapeutic agents were used simultaneously, and ten patients underwent bone marrow transplantation; thus, the association between the use of cyclophosphamide and the development of PAH was not clearly elucidated [[3], [4], [5], [6]]. In contrast to these previous reports, the only factor that could have induced PAH in this case was the use of cyclophosphamide.

Using a rabbit model, Benoit et al. demonstrated the mechanisms of cyclophosphamide-induced PH, indicating that cyclophosphamide causes medial hypertrophy of pulmonary arteries, neomuscularization of distal microvessels, congestion and hyperplasia of alveolar septa, and thickening and adventitial fibrosis of pulmonary veins [4]. They also reported that cyclophosphamide induces an elevation of pulmonary arterial pressure along with alveolar septal thickening, with accumulation of foamy intraalveolar macrophages [4]. Moreover, they showed an association between PH and an increase in plasma endothelin-1 levels, a potent vasoconstrictor involved in human PAH [4]. All these indicate that multiple mechanisms are involved in the development of cyclophosphamide-induced PH. On the other hand, most patients using cyclophosphamide do not develop PH. Rather, cyclophosphamide improves PH in some cases with inflammatory diseases, such as systemic lupus erythematosus and mixed connective tissue disease; thus, susceptibility may differ depending on the inflammatory condition and individuals [9].

Regarding the clinical course of cyclophosphamide-induced PH, two reports have documented cases with short-term use of high-dose cyclophosphamide (2700 mg/body or 7200 mg/m^2^), resulting into the development of PH within a few weeks, and consequent improvement with cessation of cyclophosphamide and corticosteroids. These reports imply that reversible mechanisms such as increased vascular tone and transient endothelial dysfunction were the primary mechanisms of pathogenesis in these cases [7,8]. In contrast, the patient in our report developed PAH after 7 years of low-dose cyclophosphamide (approximately 500 mg/week) treatment, and the patient did not fully recover, implying that more irreversible factors, such as fibrosis and/or proliferation of the vasculature, were likely to be involved. Indeed, in cases of lung injury due to cyclophosphamide, early onset lung injury at high doses is usually reversible, whereas late-onset lung injury at low doses is known to cause irreversible pulmonary fibrosis [10].

Treatment of cyclophosphamide-induced PAH first starts with the discontinuation of the drug and the addition of pulmonary vasodilators in severe cases or unstable hemodynamics [2]. Initial combination therapy with agents of different mechanisms of action is usually recommended for PAH, whereas treatment-induced pulmonary edema is a concern in PVOD [2]. In our present case, as the patient had possible PVOD, as described above, low-dose tadalafil monotherapy was initiated and the pulmonary arterial pressure and PVR were reduced without causing pulmonary edema.

In conclusion, the present case report documented a patient with RHC-proven PAH that developed after 7 years of cyclophosphamide treatment. The patient's clinical course suggested that cyclophosphamide-associated PAH was the most likely diagnosis, although the possibility of idiopathic PAH could not be ruled out. As cyclophosphamide is widely used for a variety of diseases, it should be kept in mind that cyclophosphamide can cause PAH, as a clinical phenotype of PVOD. Early recognition, prompt discontinuation of cyclophosphamide, and the use of pulmonary vasodilators need to be considered in such cases.

## 4 Conclusion

• We presented a case of PAH with a remarkably low DLCO that developed after 7 years of cyclophosphamide use.
• If cyclophosphamide is thought to induce PAH, the drug should be discontinued as soon as possible, and careful introduction of pulmonary vasodilating treatment should be considered.

## Funding

This research did not receive any specific grant from funding agencies in the public, commercial, or not-for-profit sectors.

## Data statement

Our data are unsuitable to post.

## Declaration of competing interest

The authors declare that they have no known competing financial interests or personal relationships that could have appeared to influence the work reported in this paper.
